# Evaluation of Antimicrobial Activity of Gallic Acid, Quercetin-3-D-Glucuronide, and Apigenin Against Gram-Negative Uropathogens: A Novel Approach to Urinary Tract Infection Therapy

**DOI:** 10.3390/ijms27125463

**Published:** 2026-06-17

**Authors:** Dagmara Fydrych, Jagoda Jeziurska-Pavlenko, Joanna Kwiecińska-Piróg

**Affiliations:** Department of Microbiology, Collegium Medicum of L. Rydygier in Bydgoszcz, Nicolaus Copernicus University in Toruń, 9 M. Skłodowskiej-Curie Street, 85-094 Bydgoszcz, Poland; 503577@doktorant.umk.pl (D.F.); jagoda.jeziurska@doktorant.umk.pl (J.J.-P.)

**Keywords:** urinary tract infections, gallic acid, quercetin-3-D-glucuronide

## Abstract

Urinary tract infections (UTIs), particularly catheter-associated UTIs (CAUTIs), represent a significant clinical problem due to the predominance of Gram-negative uropathogens, their ability to form biofilms, and the increasing prevalence of antimicrobial resistance, which together reduce the effectiveness of conventional antibiotic therapy. This study aimed to evaluate the antimicrobial activity of selected natural plant-derived compounds against clinical Gram-negative uropathogens isolated from CAUTIs. The antibacterial effects of gallic acid, quercetin-3-D-glucuronide, and apigenin were assessed against *Escherichia coli*, *Pseudomonas aeruginosa*, and *Proteus mirabilis*, including both ciprofloxacin-susceptible and -resistant strains. Antimicrobial activity was determined using the broth microdilution method, followed by quantitative assessment of bacterial viability based on colony-forming unit (CFU) enumeration. Gallic acid exhibited the strongest concentration-dependent inhibitory activity, reducing bacterial viability by up to 2–3 log_10_ CFU across all tested species. Quercetin-3-D-glucuronide demonstrated moderate antibacterial effects with a predominantly bacteriostatic profile, resulting in a partial but consistent reduction in CFU counts. In contrast, apigenin showed only weak effects on bacterial viability under the applied experimental conditions. None of the tested compounds achieved complete bacterial eradication. These findings indicate that gallic acid and quercetin-3-D-glucuronide possess inhibitory activity against Gram-negative uropathogens, including antibiotic-resistant strains, supporting their potential use as adjunctive agents targeting bacterial persistence in UTIs rather than as standalone antimicrobials. In the present study, the viability of planktonic bacterial cells was assessed; however, future studies should focus on evaluating the direct impact of the tested compounds on biofilm structure and biofilm formation dynamics.

## 1. Introduction

Urinary tract infections (UTIs) are among the most prevalent bacterial infections worldwide and constitute a substantial clinical, epidemiological, and economic burden on healthcare systems. They affect millions of individuals annually and are associated with significant morbidity, reduced quality of life, and high recurrence rates, particularly among women, elderly patients, and individuals requiring long-term catheterization. UTIs are predominantly caused by Gram-negative bacteria, with *Escherichia coli* being the principal etiological agent responsible for the majority of community-acquired infections. Other clinically important uropathogens include *Pseudomonas aeruginosa* and *Proteus mirabilis*, which are especially prevalent in complicated, hospital-acquired, and catheter-associated UTIs [1]. A critical factor underlying the persistence and recurrence of UTIs is the ability of uropathogens to form biofilms on uroepithelial surfaces and indwelling medical devices, particularly urinary catheters [2]. Biofilms are highly structured microbial communities embedded in a self-produced extracellular polymeric substance (EPS) matrix composed of polysaccharides, proteins, extracellular DNA, and lipids. This matrix confers substantial protection against environmental stressors, antimicrobial agents, and host immune defenses [2,3]. As a consequence, biofilm-associated bacteria display markedly increased tolerance to antimicrobial therapy, often surviving antibiotic concentrations far exceeding those required to eradicate planktonic cells [3]. These characteristics make biofilm-associated UTIs particularly difficult to treat and contribute to chronic infection and frequent relapse [4]. It is therefore essential to recognize that urinary tract infections cannot be fully understood without considering the role of biofilm formation. Among Gram-negative uropathogens, *Pseudomonas aeruginosa* is particularly notorious for its exceptional biofilm-forming capacity, extensive intrinsic and acquired antibiotic resistance, and sophisticated quorum-sensing (QS) regulatory networks. These QS systems coordinate the expression of virulence factors and biofilm maturation, enabling *P. aeruginosa* to persist in hostile environments and evade antimicrobial treatment [5,6,7,8,9]. Uropathogenic *E. coli* (UPEC) employs distinct strategies, including intracellular bacterial communities and biofilm-like aggregates, which allow long-term colonization of the urinary tract and facilitate recurrent infections [1]. *Proteus mirabilis*, in turn, is characterized by strong urease activity and swarming motility, leading to the formation of crystalline biofilms on catheter surfaces that are exceptionally resistant to antimicrobial eradication and often result in catheter blockage [6]. Collectively, these pathogens represent major therapeutic challenges in contemporary UTI management. The rapid global spread of antimicrobial resistance (AMR) among uropathogens has further complicated the treatment of UTIs and underscores the urgent need for alternative or complementary therapeutic strategies [4]. Conventional antibiotics primarily target bacterial growth or viability but often fail to eradicate biofilm-associated infections. Consequently, increasing attention has been directed toward therapeutic approaches that focus on disrupting biofilm formation, attenuating virulence, and modulating bacterial behavior rather than exerting strong bactericidal effects alone [10]. In this context, natural compounds of plant origin have attracted considerable scientific interest due to their structural diversity, pleiotropic biological activity, and comparatively low propensity to induce resistance [11]. Numerous phytochemicals have been shown to exhibit antimicrobial activity through mechanisms distinct from those of conventional antibiotics, including inhibition of bacterial adhesion, interference with quorum-sensing pathways, and modulation of EPS synthesis [10,12]. These mechanisms target conserved regulatory processes involved in biofilm development and are therefore particularly relevant for Gram-negative uropathogens. Polyphenolic compounds such as gallic acid, apigenin, and quercetin represent a particularly promising class of natural substances with documented antimicrobial activity [13]. These compounds have been extensively investigated for their potential to interfere with biofilm formation and to suppress virulence-related traits in *E. coli*, *P. aeruginosa*, and *P. mirabilis*, often at subinhibitory concentrations that may reduce selective pressure associated with resistance development [12]. Such properties are considered particularly relevant in the context of chronic and recurrent UTIs, where prolonged antibiotic exposure is common.

### 1.1. Gallic Acid

Gallic acid (3,4,5-trihydroxybenzoic acid) is a naturally occurring phenolic acid widely distributed in fruits, vegetables, tea, and medicinal plants. Although it is well known for its antioxidant and anti-inflammatory properties, increasing evidence indicates that gallic acid also exhibits significant antimicrobial and anti-biofilm activity [14]. Studies suggest that gallic acid may disrupt bacterial cell membrane integrity, induce intracellular oxidative stress, and interfere with essential metabolic processes [15]. Moreover, gallic acid has been reported to affect biofilm-related properties by limiting bacterial incorporation into biofilm structures and reducing EPS production and bacterial adhesion to abiotic and biotic surfaces [16]. These effects are particularly relevant for *P. aeruginosa* and *P. mirabilis*, whose EPS-rich biofilms play a central role in antimicrobial tolerance and catheter-associated infections.

### 1.2. Apigenin

Apigenin (4′,5,7-trihydroxyflavone) is a flavonoid commonly found in parsley, celery, chamomile, and other plant-derived foods. In addition to its antioxidant and anti-inflammatory properties, apigenin has demonstrated antimicrobial activity against a variety of Gram-negative bacteria [17]. Notably, apigenin interferes with bacterial quorum-sensing systems, leading to reduced expression of virulence factors and inhibition of biofilm development [18]. This mechanism is particularly significant in *P. aeruginosa*, whose pathogenicity is tightly regulated by QS networks. Moreover, apigenin frequently exerts its effects at sub-inhibitory concentrations, thereby minimizing selective pressure for resistance development [19]. These characteristics position apigenin as a promising anti-virulence agent in UTI therapy.

### 1.3. Quercetin

Quercetin (3,3′,4′,5,7-pentahydroxyflavone) is one of the most extensively studied flavonoids and is widely distributed in fruits, vegetables, and medicinal plants. Numerous studies have demonstrated its broad-spectrum antimicrobial activity against both Gram-positive and Gram-negative bacteria, including multidrug-resistant uropathogens [20]. The antimicrobial effects of quercetin are mediated through multiple mechanisms, such as disruption of bacterial membranes, inhibition of nucleic acid synthesis, and interference with cellular energy metabolism [21]. It has also been reported that quercetin may increase bacterial susceptibility to antimicrobial agents through interference with bacterial metabolic processes [22]. Synergistic interactions between quercetin and conventional antibiotics have also been reported, highlighting its potential role as an adjunctive agent in the treatment of UTIs caused by *E. coli* and *P. aeruginosa* [23].

Targeting biofilm formation rather than bacterial viability alone has emerged as a promising strategy in the management of chronic and recurrent UTIs [5,10]. Natural compounds such as gallic acid, apigenin, and quercetin act at multiple stages of biofilm development, including initial adhesion, maturation, and dispersion [6,8]. Their multitarget activity and ability to modulate bacterial behavior without exerting strong bactericidal effects make them attractive candidates for innovative therapeutic approaches. Recent studies, including the work of Fydrych et al. [24], further support the potential application of plant-derived compounds as effective anti-biofilm agents against clinically relevant uropathogens. The aim of the present study was to evaluate the antimicrobial activity of quercetin, apigenin, and gallic acid against selected Gram-negative uropathogens, with particular emphasis on *Escherichia coli*, *Pseudomonas aeruginosa*, and *Proteus mirabilis*. By assessing their effects on planktonic growth, this study seeks to provide a scientific basis for the potential use of natural compounds as alternative or adjunctive agents in the treatment of urinary tract infections.

## 2. Results

### 2.1. Effect of Quercetin-3-D-Glucuronide on Bacterial Viability

**Figure 1:**
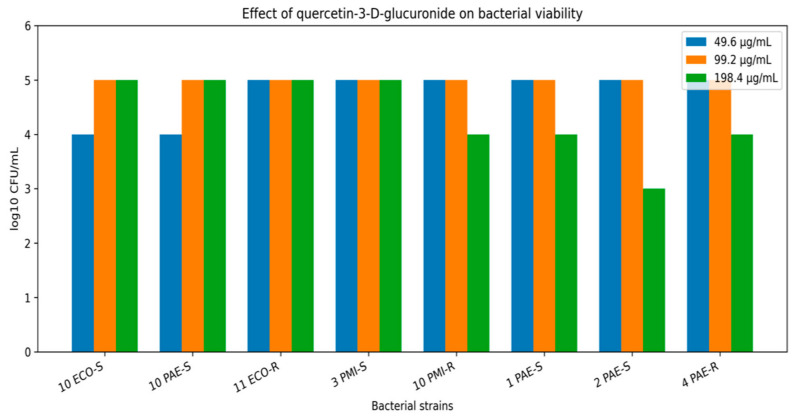
Quantitative assessment of bacterial viability following exposure to quercetin-3-D-glucuronide at concentrations ranging from 49.6 to 198.4 µg/mL. Bacterial viability was expressed as log10 CFU/mL following quantitative plating on Columbia agar supplemented with 5% sheep blood after 24 h incubation at 37 °C. The tested strains included ciprofloxacin-susceptible and ciprofloxacin-resistant clinical isolates of *Escherichia coli* (10 ECO-S, 11ECO-R), *Pseudomonas aeruginosa* (1 PAE-S, 2 PAE-S, 10 PAE-S,4 PAE-R), and *Proteus mirabilis* (3 PMI-S, 10 PMI-R). Data are presented as representative values obtained from technical replicates performed within the same experiment. A reduction in bacterial viability was observed at higher concentrations of the compound; however, complete growth inhibition was not achieved. Graphs were generated using Python (version 3.11.9; Python Software Foundation, Wilmington, DE, USA) and the Matplotlib library (version 3.8.4).

Quantitative viability assessment revealed a concentration-dependent reduction in bacterial viability following exposure to quercetin-3-D-glucuronide (Figure 1). A reduction in CFU values was observed at higher concentrations of quercetin-3-D-glucuronide; however, the effect varied between the tested strains. The inhibitory effect of quercetin-3-D-glucuronide was moderate and did not demonstrate a strictly concentration-dependent pattern for all tested isolates.

Notably, ciprofloxacin-resistant *Pseudomonas aeruginosa* strains exhibited a reduction in viability comparable to that observed for ciprofloxacin-susceptible strains, indicating that resistance to fluoroquinolones did not abolish the response to quercetin-3-D-glucuronide. Similarly, *Escherichia coli* and *Proteus mirabilis* strains showed a gradual but incomplete reduction in CFU values across the tested concentration range. Importantly, no complete bacterial eradication (0 CFU) was observed for any strain, indicating a predominantly bacteriostatic effect of quercetin-3-D-glucuronide.

### 2.2. Effect of Gallic Acid on Bacterial Viability

**Figure 2:**
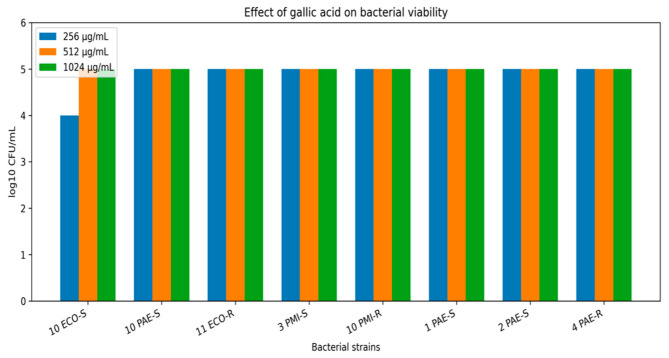
Quantitative assessment of bacterial viability following exposure to gallic acid at concentrations ranging from 256 to 1024 µg/mL. Bacterial viability was expressed as log10 CFU/mL following quantitative plating on Columbia agar supplemented with 5% sheep blood after 24 h incubation at 37 °C. The tested strains included ciprofloxacin-susceptible and ciprofloxacin-resistant clinical isolates of *Escherichia coli* (10 ECO-S, 11 ECO-R), *Pseudomonas aeruginosa* (1 PAE-S, 2 PAE-S, 10 PAE-S, 4 PAE-R), and *Proteus mirabilis* (3 PMI-S, 10 PMI-R). Data are presented as representative values obtained from technical replicates performed within the same experiment. A reduction in bacterial viability was observed with increasing concentrations of gallic acid; however, complete bacterial growth inhibition was not achieved under the tested conditions. Graphs were generated using Python (version 3.11.9; Python Software Foundation, Wilmington, DE, USA) and the Matplotlib library (version 3.8.4).

Exposure to gallic acid resulted in a noticeable reduction in bacterial viability in several tested strains (Figure 2). Higher concentrations of gallic acid were generally associated with lower CFU values; however, variability between strains was observed. The magnitude of reduction varied between strains but was consistently observed for *P. aeruginosa*, *E. coli*, and *P. mirabilis*, regardless of their ciprofloxacin susceptibility profiles.

At lower concentrations, the inhibitory effect was less pronounced and differed depending on the bacterial isolate. Despite the pronounced reduction in CFU counts, viable bacteria were still detectable, confirming that gallic acid exerted a strong inhibitory but non-bactericidal effect under the experimental conditions.

### 2.3. Effect of Apigenin on Bacterial Viability

**Figure 3:**
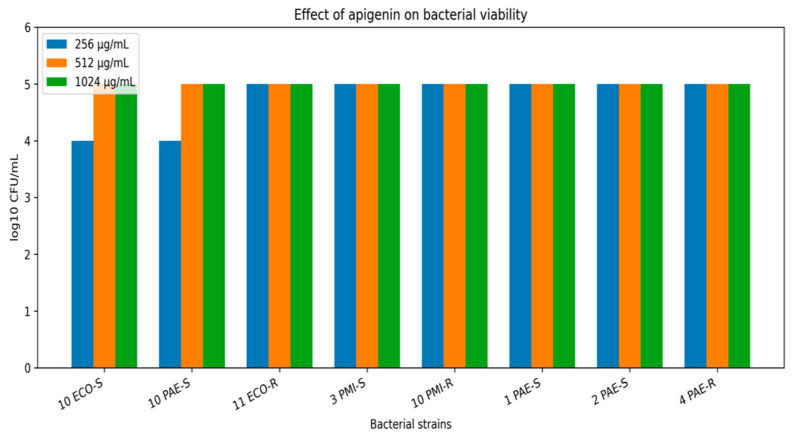
Quantitative assessment of bacterial viability following exposure to apigenin at concentrations ranging from 256 to 1024 µg/mL. Bacterial viability was expressed as log10 CFU/mL following quantitative plating on Columbia agar supplemented with 5% sheep blood after 24 h incubation at 37 °C. The tested strains included ciprofloxacin-susceptible and ciprofloxacin-resistant clinical isolates of *Escherichia coli* (10 ECO-S, 11ECO-R), *Pseudomonas aeruginosa* (1 PAE-S, 2 PAE-S, 10 PAE-S,4 PAE-R), and *Proteus mirabilis* (3 PMI-S, 10 PMI-R). Data are presented as representative values obtained from technical replicates performed within the same experiment. Only a limited reduction in bacterial viability was observed, and no complete growth inhibition was detected under the tested conditions. Graphs were generated using Python (version 3.11.9; Python Software Foundation, Wilmington, DE, USA) and the Matplotlib library (version 3.8.4).

In contrast to quercetin-3-D-glucuronide and gallic acid, apigenin demonstrated a weaker impact on bacterial viability (Figure 3). At higher concentrations (1024 and 512 µg/mL), only a moderate reduction in CFU values was observed for selected strains, while for others the effect was minimal. At the lowest tested concentration (256 µg/mL), CFU values were largely comparable to those of the growth control.

Due to the limited inhibitory activity observed at higher concentrations, additional viability assessment was performed after further dilution of apigenin (64, 32, and 16 µg/mL). However, even after this adjustment, no substantial reduction in CFU counts was detected. Overall, apigenin displayed the lowest antibacterial activity among the tested compounds and did not induce a pronounced concentration-dependent decrease in bacterial viability.

**Figure 4:**
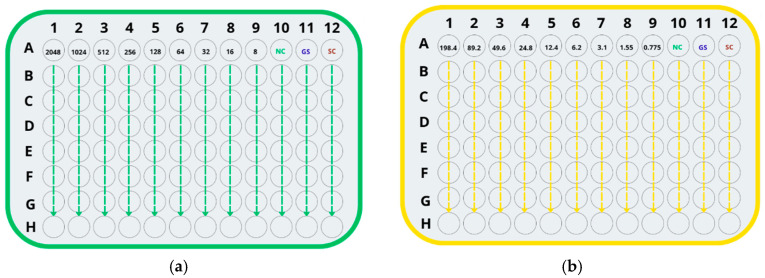
(**a**) Schematic layout of the 96-well microtiter plate used for gallic acid dilution series preparation. (**b**) Schematic layout of the 96-well microtiter plate used for apigenin dilution series preparation. Figures were prepared using Canva (Canva Pty Ltd., Sydney, Australia).

Schematic representation of sample distribution in a 96-well polystyrene microtiter plate used for the determination of the minimum inhibitory concentration (MIC) of gallic acid and apigenin using the broth microdilution serial dilution method (Figure 4). Two-fold serial dilutions (1:2) of the tested compounds were prepared from left to right (columns 1–9), resulting in decreasing concentrations. All wells within a single column contained the same concentration of the tested compound and served as technical replicates. Column 10 corresponded to the negative control (NC), column 11 to the growth control (GC), and column 12 to the solvent control (SC).

### 2.4. Quantitative Viability Assessment Following MIC Determination

**Figure 5:**
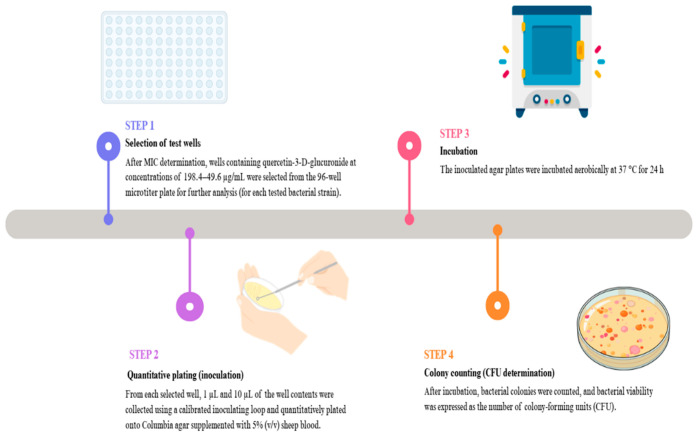
Quantitative viability assessment following MIC determination. Prepared in Canva (Sydney, Australia).

To assess bacterial viability following exposure to quercetin-3-D-glucuronide, for each tested strain, samples were collected from wells of the 96-well microtiter plate containing concentrations ranging from 198.4 to 49.6 µg/mL. In the case of gallic acid and apigenin, samples were collected from wells containing concentrations of 1024, 512, and 128 µg/mL. From each selected well, 1 µL and 10 µL aliquots were collected using calibrated disposable inoculation loops and subsequently plated on Columbia agar supplemented with 5% (*v*/*v*) sheep blood for quantitative viability assessment. Due to the lack of a potentially inhibitory effect on bacterial growth observed for apigenin, an additional dilution step was applied prior to viability assessment. The contents of selected wells containing apigenin at concentrations of 64, 32, and 16 µg/mL were further diluted 1:1 in sterile physiological saline (0.9% NaCl). Following this additional dilution, quantitative plating was performed using a calibrated 10 µL inoculation loop, with samples collected from the diluted wells (wells 6–10). The agar plates were incubated aerobically at 37 °C for 24 h. After incubation, bacterial colonies were counted, and bacterial viability was expressed as colony-forming units (CFU) (Figure 5).

## 3. Discussion

The present study evaluated the antimicrobial activity of selected natural compounds—gallic acid, quercetin-3-D-glucuronide, and apigenin—against Gram-negative uropathogens associated with catheter-associated urinary tract infections. The obtained results revealed clear differences in the inhibitory potential of the tested phytochemicals, with gallic acid demonstrating the strongest antibacterial effect, quercetin-3-D-glucuronide showing moderate activity, and apigenin exhibiting the weakest impact on bacterial viability under the applied experimental conditions.

The pronounced antibacterial activity of gallic acid observed in this study is consistent with earlier reports describing phenolic acids as effective antimicrobial agents against Gram-negative bacteria [12,14,15,16,25]. Previous studies have demonstrated that gallic acid disrupts bacterial cell membrane integrity, increases membrane permeability, and induces oxidative stress, leading to impaired cellular homeostasis and reduced bacterial survival [14,25]. In addition, gallic acid has been shown to significantly reduce bacterial adhesion on abiotic surfaces, which is of particular importance in catheter-associated urinary tract infections [6,16].

Quercetin and its derivatives are among the most extensively studied flavonoids with documented antimicrobial properties [12,13,21,22,23]. Previous studies suggest that quercetin may interfere with nucleic acid synthesis, alter membrane function, and reduce the production of extracellular polymeric substances, which may affect the incorporation of bacterial cells into the biofilm matrix [21,22].

Moreover, quercetin has been reported to modulate quorum–sensing-regulated gene expression, leading to attenuation of virulence without complete bacterial killing [14,23]. The moderate reduction in bacterial viability observed in the present study is generally consistent with the predominantly bacteriostatic mode of action described in the literature.

In contrast, apigenin exhibited limited antibacterial activity under the applied experimental conditions. Although previous studies have suggested that apigenin may interfere with quorum-sensing systems and biofilm-related processes in Gram-negative bacteria, its direct antimicrobial activity appears to be weaker than that reported for phenolic acids and other flavonoids [17,18,19]. Earlier reports indicate that apigenin may primarily modulate bacterial communication and gene regulation rather than strongly inhibit bacterial growth [18]. In the present study, no clear effect of apigenin on bacterial viability was observed. Therefore, further investigations are required to better characterize its potential role in bacterial cell communication and biofilm-related processes.

From a clinical perspective, the obtained results support the concept that targeting bacterial biofilm formation and virulence regulation, rather than bacterial viability alone, represents a promising complementary strategy in the management of chronic and recurrent urinary tract infections [5,10,26]. Gram-negative uropathogens such as *Escherichia coli*, *Pseudomonas aeruginosa*, and *Proteus mirabilis* are well known for their ability to form biofilms and persist in the urinary tract despite antibiotic therapy [1,2,5]. Natural compounds with anti-biofilm or anti-virulence activity may therefore serve as adjunctive agents that enhance the effectiveness of conventional antimicrobial treatments and potentially reduce selective pressure for resistance development [9,10].

Nevertheless, caution must be exercised when extrapolating in vitro findings to clinical applications. Previous studies have emphasized that effective concentrations observed in vitro may not be readily achievable in vivo and that compound activity may be significantly influenced by environmental conditions, compound stability, and host factors [10,24]. Consequently, further studies are required to elucidate detailed mechanisms of action, evaluate synergistic effects with antibiotics, and assess therapeutic relevance using advanced biofilm and in vivo models.

## 4. Materials and Methods

### 4.1. Materials

The epidemiological analysis was conducted using the Promic software database (Mori®, Marcin Bogucki, Bydgoszcz, Poland). The study included a total of eight clinical Gram-negative bacterial isolates associated with catheter-associated urinary tract infections (CAUTIs).

The strain panel consisted of two *Escherichia coli* isolates, including one ciprofloxacin-susceptible strain and one ciprofloxacin-resistant strain. In addition, four *Pseudomonas aeruginosa* isolates were analyzed, comprising three ciprofloxacin-susceptible strains and one ciprofloxacin-resistant strain. Furthermore, two *Proteus mirabilis* isolates were included, one ciprofloxacin-susceptible and one ciprofloxacin-resistant strain.

All bacterial strains were isolated from patients with microbiologically confirmed CAUTIs who were treated at the University Hospital No. 1, named after Dr. Antoni Jurasz, in Bydgoszcz, Poland.

The clinical isolates used in this study are part of the strain collection of the Department of Microbiology, Collegium Medicum Ludwik Rydygier in Bydgoszcz, Nicolaus Copernicus University in Toruń. All bacterial strains were stored at −80 °C in a freezer (Thermo Fisher Scientific, Waltham, MA, USA) in tryptic soy broth (Becton, Dickinson and Company, Franklin Lakes, NJ, USA) supplemented with 20% (*v*/*v*) glycerol (Avantor Performance Materials, Gliwice, Poland) until further use.

### 4.2. Methods

#### 4.2.1. Determination of Bacterial Cell Viability

The investigated bacterial strains were recovered from frozen stocks by subculturing using the streak plate method onto Columbia agar supplemented with 5% (*v*/*v*) sheep blood (Becton Dickinson). Plates were incubated aerobically at 37 °C for 24 h. From 24-h cultures, bacterial suspensions were prepared in sterile 0.9% (*w*/*v*) sodium chloride solution and adjusted to a turbidity of 0.5 McFarland, corresponding to approximately 1 × 10^8^ CFU/mL. These standardized suspensions were used as inocula for subsequent experiments. *Escherichia coli* ATCC 25922 was used as the reference control strain for quality control of antimicrobial susceptibility testing.

Sterile 96-well polystyrene microtiter plates were prepared by dispensing 178 µL of Mueller–Hinton broth (MHB; Becton Dickinson) into each well. Stock solutions of the tested phytochemicals were prepared using analytical-grade compounds purchased from pol-AURA (Dywity, Poland) and subsequently diluted in Mueller–Hinton broth.

Two-fold serial dilutions (1:2) of each compound were prepared in Mueller–Hinton broth (MHB) to obtain the required concentration ranges. The prepared dilution series was subsequently transferred to sterile 96-well microtiter plates according to the experimental design.

The serial dilution schemes applied in the microtiter plates are schematically illustrated in Figure 1 for quercetin-3-O-glucuronide and in Figure 2 for gallic acid and apigenin.

For each well, 20 µL of the appropriate phytochemical dilution was added, followed by 2 µL of the standardized bacterial suspension, resulting in a final volume of 200 µL per well. The final concentrations of the tested compounds were obtained after the addition of all experimental components to the wells. Based on commonly applied in vitro protocols, the final concentration of ethanol in the wells did not exceed 1% (*v*/*v*), which is generally considered non-inhibitory for bacterial growth under standard in vitro conditions.

MIC values of the tested phytochemicals were determined using the broth microdilution method in 96-well polystyrene microtiter plates.

Throughout all experiments, negative controls (NC), growth controls (GC), and solvent controls (SC) were included. All experiments were performed under aseptic conditions Table 1.

#### 4.2.2. Statistical Analysis

The study was conducted as a preliminary exploratory investigation using a limited number of clinical isolates (*n* = 8). Quantitative viability assessment was performed using technical replicates within the same experimental setup. Due to the descriptive character of the study and the absence of independent biological replicates, formal inferential statistical analyses were not applied. Consequently, error bars and statistical annotations were not included in the figures. Therefore, the obtained results are presented descriptively as log10 CFU/mL values and should be interpreted as preliminary observations requiring further validation using larger strain collections and independent biological replicates.

## 5. Conclusions

Gallic acid exhibited the strongest inhibitory effect against Gram-negative uropathogens among the tested phytochemicals, followed by quercetin-3-D-glucuronide, while apigenin showed the weakest antibacterial activity.

The observed effects are consistent with previous reports indicating that phenolic acids and selected flavonoids reduce bacterial viability and modulate biofilm-related processes [5,6,12,13,14,15,16,21,22,23,25].

None of the tested compounds induced complete bacterial eradication under the applied experimental conditions, which may suggest a predominantly growth-inhibitory effect, as previously described for many natural antimicrobial agents [10,12,26]. The results support the potential application of natural compounds as adjunctive agents in antimicrobial therapy, particularly in strategies aimed at limiting biofilm-associated infections and bacterial persistence [5,9,10].

Further investigations employing expanded strain collections, biofilm-specific experimental models, and combination therapies with antibiotics are required to fully assess the clinical relevance of these findings.

## Figures and Tables

**Table 1:** Composition of control and test wells.

Well Type	MHB ^V^ [μL]	Phytochemical ^V^ [μL]	Bacterial Suspension ^V^ [μL]	Ethanol Content [%]	Description
NC	200	-	-	-	Medium sterility control
GC	198	-	2	-	100% bacterial growth
SC	178	20 *	2	≤1% (*v*/*v*)	Solvent control
Test well	178	20	2	≤1% (*v*/*v*)	MIC determination

^V^—volume of solution, * 20 µL of MHB containing ethanol at the same final concentration as in test wells.

## Data Availability

The original contributions presented in this study are included in the article. Further inquiries can be directed to the corresponding authors.
